# Effects of Bt cabbage pollen on the honeybee *Apis mellifera* L

**DOI:** 10.1038/s41598-017-18883-w

**Published:** 2018-01-11

**Authors:** Dengxia Yi, Zhiyuan Fang, Limei Yang

**Affiliations:** 1 Institute of Animal Science, Chinese Academy of Agricultural Sciences, Beijing, 100193 China; 2 Key Laboratory of Biology and Improvement of Horticultural Crops, Ministry of Agriculture, Institute of Vegetables and Flowers, Chinese Academy of Agricultural Sciences, Beijing, 100081 China

## Abstract

Honeybees may be exposed to insecticidal proteins from transgenic plants via pollen during their foraging activity. Assessing effects of such exposures on honeybees is an essential part of the risk assessment process for transgenic *Bacillus thuringiensis* (Bt) cabbage. Feeding trials were conducted in a laboratory setting to test for possible effects of Cry1Ba3 cabbage pollen on Italian-derived honeybees *Apis mellifera* L. Newly emerged *A*. *mellifera* were fed transgenic pollen, activated Cry1Ba3 toxin, pure sugar syrup (60% w/v sucrose solution), and non-transgenic cabbage pollen, respectively. Then the effects on survival, pollen consumption, weight, detoxification enzyme activity and midgut enzyme activity of *A*. *mellifera* were monitored. The results showed that there were no significant differences in survival, pollen consumption, weight, detoxification enzyme activity among all treatments. No significant differences in the activities of total proteolytic enzyme, active alkaline trypsin-like enzyme and weak alkaline trypsin-like enzyme were observed among all treatments. These results indicate that the side-effects of the Cry1Ba3 cabbage pollen on *A*. *mellifera* L. are unlikely.

## Introduction

Genetic engineering has been successfully applied in many crop breeding programs, and 2 billion hectares of transgenic crops were successfully cultivated globally from 1996 to 2015^1^. The planting of transgenic crops has increased resistance by the target pests, but reduced the use of chemical pesticides, and produced great economic and social benefits^1,2^. However, the worldwide planting of transgenic crops has triggered concerns about their potential effects on non-target organisms^3–5^, such as honeybees. Honeybees are economically valuable pollinators that are essential for seed production of many crops and wild plants. They can also maintain ecological balance via pollination. Honeybees may be exposed to insecticidal proteins in the pollen from transgenic plants, therefore, they are considered as an important non-target organism in the biosafety assessment of transgenic crops^3,6^.

Multiple studies have been conducted to evaluate the effects of transgenic products on honeybees^6–13^. Adult *Apis mellifera* L. fed on transgenic corn pollen (containing *cry1Ab*) mixed thoroughly into sugar syrup showed no significant differences in survival and hypopharyngeal gland growth compared with controls after 10 days^14^. No significant differences were detected in the pollen consumption and hypopharyngeal gland weight of *A*. *mellifera* and *Apis cerana cerana* worker honeybees fed on sugar syrup containing the Cry1Ah toxin compared with the control^15^, and transgenic Cry1Ah maize pollen did not affect the midgut communities in larvae and adult honeybees^16^. In addition to the growth and survival rate of honeybees, pupal dry weight^17^, longevity and food consumption rate^18–20^, mortality^21,22^, flight activity^23^, foraging activity and learning performance^10,20^, cap rate and emergence rate^24^, as well as feeding behaviour^25^ have also been studied, and overall these results indicate that Bt toxins have no negative effect on honeybees.

Midgut and detoxification enzymes are important parameters that should be evaluated as part of the risk assessment necessary for the commercialization of *Bacillus thuringiensis* (Bt) transgenic crops^11,26^. Midgut enzymes play an important role in the digestion process of pollen ingested by honeybees^27^. The total midgut proteolytic enzyme activity is directly related to the ability to digest protein-rich pollen and may be used to assess digestion^28^. Furthermore, midgut protein digestion is associated with the development of hypopharyngeal gland and the production of extractable proteins occurs in the hypopharyngeal gland when honeybees are fed on pollen^28^. Sagili *et al*.^28^ reported that honeybees fed on pollen containing 1% soybean trypsin inhibitor had significantly reduced midgut proteolytic enzyme activity. Detoxification enzymes, such as α-naphthylacetate esterase, glutathione-S-transferase, and acetylcholinesterase, catalyze metabolic reactions that convert foreign compounds into forms that can be excreted from the body^26^.

The Bt *cry1Ba3* gene was cloned by the Institute of Plant Protection, Chinese Academy of Agriculture Sciences^29^. We previously incorporated a synthetic *cry1Ba3* gene into the genome of an elite inbred cabbage line A21–3 *via Agrobacterium tumefaciens*-mediated transformation method to produce fertile transgenic plants^29^. Insect bioassays indicated that expressing *cry1Ba3* in transgenic cabbage plants effectively controlled both susceptible and Cry1Ac-resistant *Plutella xylostella* larvae in the laboratory^29^. A healthy honeybee hive relies on landscapes with ample and nutritious sources of pollen yielding flowers. Cabbage’s flowers are bright, yellow, fragrant and attractive to honeybees. It was reported that pollen from Brassicaceae plants, including cabbages, made up about 4.89%-12.62% of all pollen collected by honeybees during the blooming period^30^. Honeybees could be easily exposed to insecticidal proteins from Bt cabbage flowers during foraging. It is important to assess the non-target effects of transgenic cabbage pollen on honeybees before its commercial release. The objective of this study was, therefore, to examine the effects of Cry1Ba3 cabbage pollen on the survival, pollen consumption, weight, and enzyme activities of worker honeybees (*A*. *mellifera*).

## Results

### Concentration of Cry1Ba3 protein in Bt cabbage pollen

The content of Cry1Ba3 protein in transgenic cabbage pollen was 778.5 ± 16.22 ng/g (mean ± SE). This pollen was used in the following experiments.

### Survival, pollen consumption and body weight

**Figure 1:**
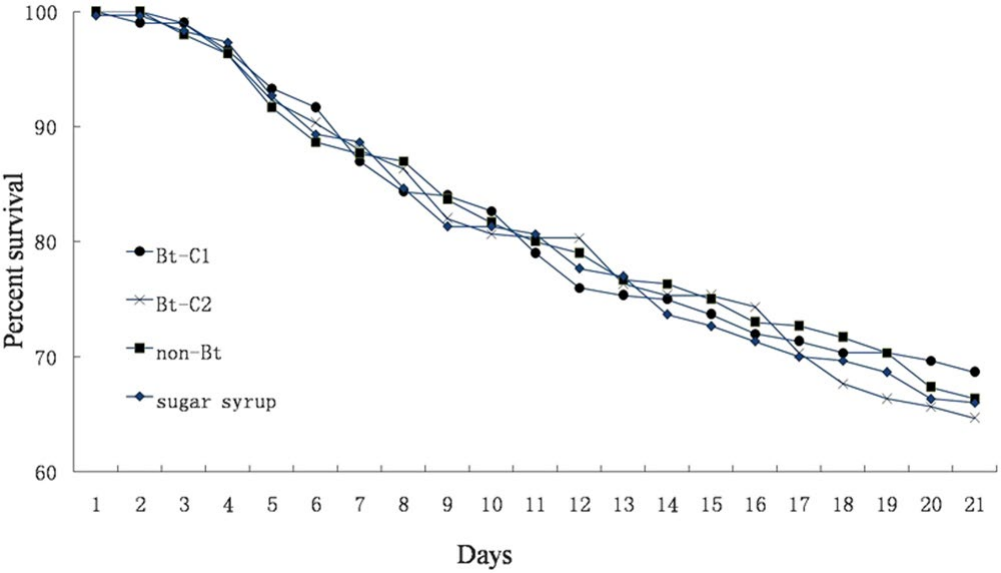
Survival of *A*. *Mellifera* fed with Bt-C1, Bt-C2, non-Bt pollen and pure sugar syrup for 21 days. The percentage of the initial number of honeybees surviving at each day after the start of treatment is shown.

The survival rate of *A*. *mellifera* did not significantly differ among the honeybees that were fed on Bt-C1, Bt-C2, non-Bt, and sugar syrup at any time point during the 21 days of the experiment (Fig. 1; Table 1). The average survival rate on day 21 for the honeybees exposed to Bt-C1, Bt-C2, non-Bt and sugar syrup was 68.7%, 64.6%, 66.3% and 66.0%, respectively and there were no significant differences among all treatments (*F* = 0.66, *df* = 23, *P* = 0.59; Fig. 1). Moreover, no significant differences were found in the pollen consumption of *A*. *Mellifera* fed on Bt-C1 and Bt-C2 compared with the control groups at any time point (Table 2). The body weight of *A*. *Mellifera* also did not differ significantly among Bt-C1, Bt-C2, non-Bt pollen and sugar syrup on days 7 (*F* = 1.14, *df* = 23, *P* = 0.36; Table 3), 14 (*F* = 2.05, *df* = 23, *P* = 0.14; Table 3) and 21 (*F* = 1.93, *df* = 23, *P* = 0.16; Table 3).

**Table 1:** Mean survival rate of *A*. *Mellifera* subjected to chronic exposure to Bt-C1, Bt-C2, non-Bt pollen and pure sugar syrup during a 21-day oral exposure.

Days	Survival rate (mean ± SE)				*F*	*df*	*P*
	Bt-C1	Bt-C2	non-Bt	sugar syrup			
1	1.000 ± 0.000	1.000 ± 0.000	1.000 ± 0.000	0.997 ± 0.001	1	23	0.41
2	0.990 ± 0.002	1.000 ± 0.000	1.000 ± 0.000	0.997 ± 0.001	2.86	23	0.06
3	0.990 ± 0.002	0.990 ± 0.003	0.980 ± 0.004	0.983 ± 0.003	0.47	23	0.70
4	0.967 ± 0.003	0.963 ± 0.003	0.963 ± 0.003	0.973 ± 0.002	0.65	23	0.60
5	0.933 ± 0.003	0.923 ± 0.003	0.917 ± 0.004	0.927 ± 0.002	0.88	23	0.47
6	0.917 ± 0.003	0.903 ± 0.004	0.887 ± 0.002	0.893 ± 0.004	2.79	23	0.07
7	0.870 ± 0.003	0.880 ± 0.003	0.877 ± 0.003	0.887 ± 0.003	0.72	23	0.50
8	0.843 ± 0.004	0.863 ± 0.005	0.870 ± 0.003	0.847 ± 0.003	1.74	23	0.19
9	0.840 ± 0.003	0.820 ± 0.004	0.837 ± 0.004	0.813 ± 0.003	2.24	23	0.11
10	0.827 ± 0.003	0.807 ± 0.003	0.817 ± 0.004	0.813 ± 0.003	1.11	23	0.37
11	0.790 ± 0.003	0.803 ± 0.004	0.800 ± 0.003	0.807 ± 0.002	0.88	23	0.47
12	0.763 ± 0.006	0.803 ± 0.004	0.790 ± 0.002	0.777 ± 0.004	2.96	23	0.06
13	0.753 ± 0.003	0.763 ± 0.005	0.767 ± 0.002	0.770 ± 0.003	0.68	23	0.58
14	0.750 ± 0.003	0.753 ± 0.007	0.763 ± 0.001	0.737 ± 0.006	0.90	23	0.46
15	0.737 ± 0.003	0.753 ± 0.007	0.750 ± 0.003	0.727 ± 0.005	1.17	23	0.35
16	0.720 ± 0.002	0.743 ± 0.007	0.730 ± 0.003	0.713 ± 0.003	1.43	23	0.26
17	0.713 ± 0.003	0.703 ± 0.003	0.727 ± 0.003	0.700 ± 0.003	2.46	23	0.09
18	0.703 ± 0.004	0.677 ± 0.005	0.717 ± 0.004	0.697 ± 0.003	2.84	23	0.06
19	0.703 ± 0.004	0.663 ± 0.006	0.703 ± 0.006	0.687 ± 0.005	2.16	23	0.12
20	0.697 ± 0.006	0.657 ± 0.007	0.673 ± 0.009	0.663 ± 0.006	1.00	23	0.41
21	0.687 ± 0.008	0.647 ± 0.0009	0.663 ± 0.010	0.660 ± 0.006	0.66	23	0.59

**Table 2:** Mean three-day cumulative quantify of food consumed (±SE) by *A*. *Mellifera* subjected to chronic exposure to Bt-C1, Bt-C2, non-Bt pollen and pure sugar syrup during a 21-day oral exposure.

Days	Pollen consumption (mg) per bee (mean ± SE)				*F*	*df*	*P*
	Bt-C1	Bt-C2	non-Bt	sugar syrup			
1–3	9.81 ± 0.35	9.22 ± 0.34	9.75 ± 0.32	9.18 ± 0.33	0.17	23	0.91
4–6	9.40 ± 0.37	9.65 ± 0.37	9.53 ± 0.38	8.88 ± 0.28	0.16	23	0.92
7–9	8.00 ± 0.31	7.90 ± 0.36	7.55 ± 0.35	7.41 ± 0.34	0.11	23	0.95
10–12	6.93 ± 0.30	7.91 ± 0.31	7.02 ± 0.28	6.59 ± 0.35	0.56	23	0.65
13–15	5.57 ± 0.41	4.49 ± 0.25	4.92 ± 0.30	5.26 ± 0.29	0.35	23	0.79
16–18	4.45 ± 0.25	3.25 ± 0.20	3.50 ± 0.33	3.41 ± 0.18	0.81	23	0.50
19–21	1.85 ± 0.15	1.85 ± 0.22	1.73 ± 0.20	1.48 ± 0.15	0.15	23	0.93
Sum	46.01 ± 0.49	44.27 ± 0.74	44.00 ± 0.58	42.20 ± 0.88	0.85	23	0.48

**Table 3:** Body weight of *A*. *Mellifera* fed with Bt-C1, Bt-C2, non-Bt pollen and pure sugar syrup for 21 days.

Days	Body weight (mg, mean ± SE)				*F*	*df*	*P*
	Bt-C1	Bt-C2	non-Bt	sugar syrup			
7	94.59 ± 0.99	92.07 ± 1.67	97.12 ± 1.30	88.53 ± 1.54	1.14	23	0.36
14	128.38 ± 1.82	138.07 ± 1.04	135.27 ± 0.95	132.52 ± 0.52	2.05	23	0.14
21	117.59 ± 1.16	107.43 ± 1.25	112.93 ± 0.87	111.95 ± 1.53	1.93	23	0.16

### Assessment of detoxification enzyme activity

After 7 days of feeding, the activities of α-naphthylacetate esterase (*F* = 0.63, *df* = 11, *P* = 0.62), glutathione-S-transferase (*F* = 0.70, *df* = 11, *P* = 0.58), and acetylcholinesterase (*F* = 0.13, *df* = 11, *P* = 0.94) in *A*. *Mellifera* fed on Bt-C1 and Bt-C2 were not significantly different from the control groups fed on non-Bt pollen or sugar syrup (Table 4).

**Table 4:** The activities of three detoxification enzymes in *A*. *Mellifera* fed with Bt-C1, Bt-C2, non-Bt pollen and pure sugar syrup for 7 days.

Detoxification enzyme	Enzyme activity (mmol·L^−1^·mg^−1^·min^−1^)				*F*	*df*	*P*
	Bt-C1	Bt-C2	non-Bt	sugar syrup			
Acetylcholinesterase	0.055 ± 0.001	0.052 ± 0.002	0.052 ± 0.003	0.053 ± 0.003	0.13	11	0.94
Glutathione-S-transferase	0.015 ± 0.000	0.016 ± 0.001	0.014 ± 0.001	0.013 ± 0.001	0.70	11	0.58
α-naphthylacetate esterase	0.034 ± 0.002	0.040 ± 0.003	0.037 ± 0.003	0.035 ± 0.001	0.63	11	0.62

### Assessment of midgut enzyme activity

**Figure 2:**
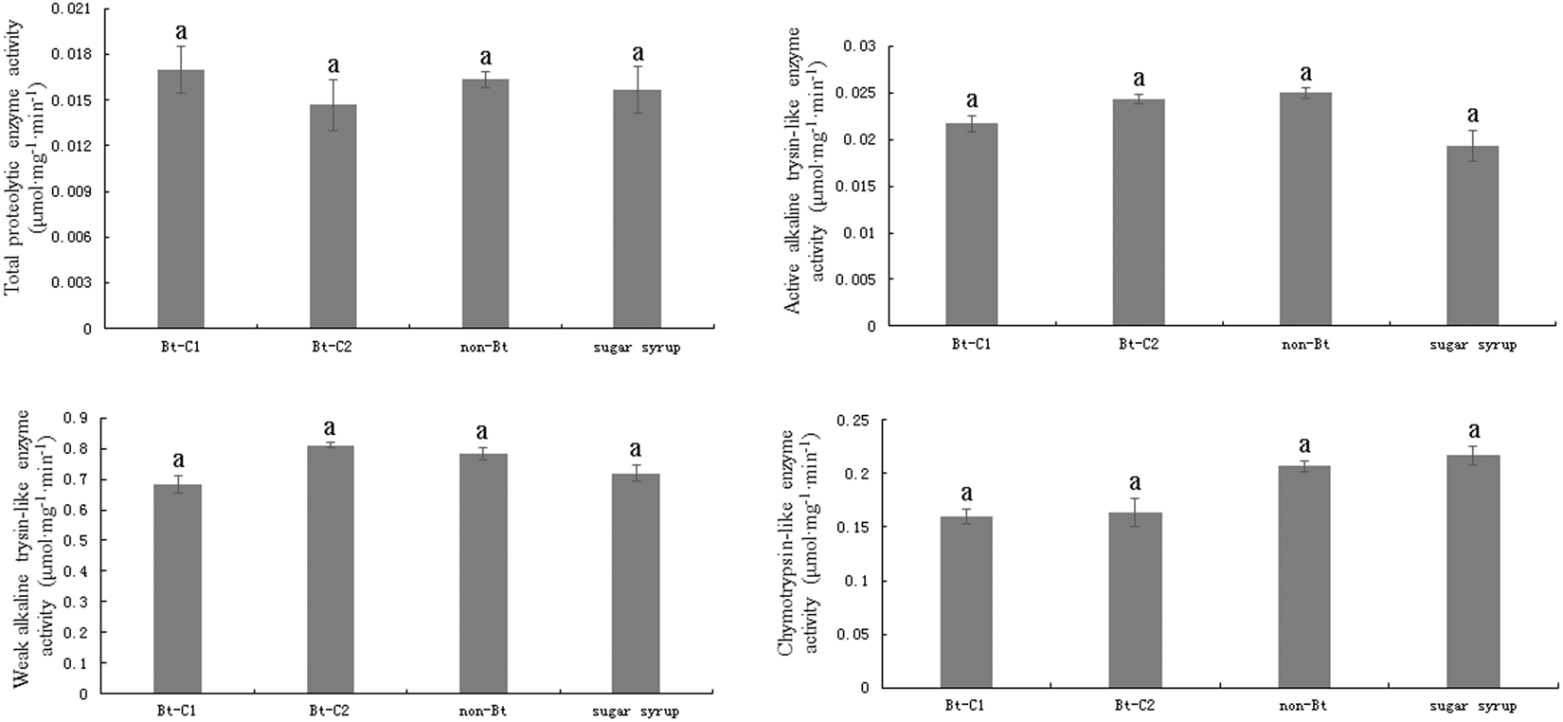
The activities of total proteolytic enzyme (n = 30), active alkaline trypsin-like enzyme (n = 30), weak alkaline trypsin-like enzyme (n = 30) and chymotrypsin-like enzyme (n = 30) in *A*. *Mellifera* fed with Bt-C1, Bt-C2, non-Bt pollen and pure sugar syrup for 7 days.

The values of midgut enzyme activity in honeybees fed on different foods are shown in Fig. 2. No significant differences in total proteolytic enzyme activity (*F* = 0.17, *df* = 11, *P* = 0.91; Fig. 2), active alkaline trypsin-like enzyme activity (*F* = 2.26, *df* = 11, *P* = 0.16; Fig. 2) and weak alkaline trypsin-like enzyme activity (*F* = 2.13, *df* = 11, *P* = 0.17; Fig. 2) were observed among all treatments, respectively. However, honeybees that were fed on Bt-C1 or Bt-C2 showed slightly lower values of chymotrypsin-like enzyme activity (*F* = 3.79, *df* = 11, *P* = 0.059; Fig. 2). But considering that the sample size is small (n = 30), the lack of statistical significance is marginal.

## Discussion

In this study, the effects of Cry1Ba3 cabbage pollen on survival, pollen consumption, weight, detoxification enzyme activity and midgut enzyme activity of *A*. *mellifera* were evaluated. The results suggest that transgenic Bt cabbage pollen carries no risk for *A*. *Mellifera* and are consistent with previous reports^6–13^. Although a slight decrease in the value of chymotrypsin-like enzyme activity was observed, the potential side effects of Cry1Ba3 cabbage pollen on the honeybee *A*. *mellifera* were limited (*F* = 3.79, *df* = 11, *P* = 0.059; Fig. 2). The midgut enzyme activity (total proteolytic enzyme, active alkaline trypsin-like enzyme weak alkaline trypsin-like enzyme, and chymotrypsin-like enzyme), which are directly related to digestive capacity of protein-rich pollen, could be sensitive indicator for assessing the development of honeybee hypopharyngeal gland^28^. A significantly decrease in the value of chymotrypsin-like enzyme activity may imply that the development of honeybee hypopharyngeal gland were influenced when fed on transgenic pollen.

In the present study, *A*. *mellifera* exposed to Bt cabbage pollen or Bt toxin showed slightly lower values of chymotrypsin-like enzyme activity, although the corresponding activity was not significantly lower than the control groups (*F* = 3.79, *df* = 11, *P* = 0.059; Fig. 2). In assessing of chymotrypsin-like enzyme activity, ten in total honeybees from each treatment were used in each replicate and three replicates were undertaken. The sample size was 30 (n = 30). In the experimental protocols reported by Han *et al*.^11^, laboratory studies to measure midgut enzyme activity tested 120 honeybees. Their results showed no side effects of CCRI41 cotton pollen on total midgut proteolytic enzyme activity of honeybees when they were subjected to chronic exposure to the transgenic CCRI41 cotton pollen. Our findings were consistent with the report, thus our results can be considered as valid and reliable. Taking the previous protocols^11^ into consideration, the sample size used in our study was relatively small. To ensure the precision, a large sample size would be required in further studies.

In this study, both Bt cabbage pollen and Bt toxin were used. The higher concentration of Cry1Ba3 toxin (Bt-C2) used is unlikely to be encountered by honeybees in the field and hence represents a worst case scenario. The lower concentration of Cry1Ba3 toxin (Bt-C1) represents a value closer to that in the field if it is expressed in the pollen. It must be noted that our study was conducted in a laboratory setting, and the results are preliminary. Some other parameters including foraging activity, learning performance, the time of first flight and the duration of flight activity should be investigated in future studies. In addition to laboratory feeding of the honeybees, field studies are also important for understanding the effects of transgenic plants on non-target organisms^31,32^. Future research should be conducted on the joint effects of transgenic Bt cabbage on honeybees in the field.

## Materials and Methods

### Cabbage pollen

The transgenic cabbage inbred line A21–3 containing the synthetic Bt *cry1Ba3* gene was produced by Yi *et al*.^29^. Non-transgenic A21–3 was used as the control. Bt and control cabbages were planted in a greenhouse belonging to the Institute of Vegetables and Flowers, Chinese Academy of Agricultural Sciences. Routine management was carried out and pesticide applications were avoided. Bt and control cabbage pollen were collected using a multi-point field sampling method at the stage of full bloom. Samples were stored at −80 °C in a refrigerator until they were used for experiments.

### Quantitative detection of Cry1Ba3 protein in Bt cabbage pollen

Quantitative determination of Cry1Ba3 protein in transgenic cabbage pollen was performed using an enzyme-liked immunosorbent assay (ELISA) kit provided by the State Key Laboratory for Biology of Plant Diseases and Insect Pests, Institute of Plant Protection, Chinese Academy of Agricultural Sciences. ELISA was carried out according to a previously published method^29,33^. Cry1Ba3 was purified from transgenic pollen using the following procedure. The pollen was homogenized in 2 ml extraction phosphate buffered saline with tween-20 (PBST; 8.0 g NaCl, 2.7 g Na_2_HPO_4_·12H_2_O, 0.4 g NaH_2_PO_4_·2H_2_O, dissolved in 1000 ml water, pH = 7.4). Then the sample was washed with 2 ml PBST and kept in a 10 ml centrifuge tube at 4 °C overnight to extract the insecticidal protein. The tube was then centrifuged at 5000 rpm for 20 min. The insecticidal protein content in the supernatant was quantified using the ELISA kit as described by Yi *et al*.^29^.

### Honyebees and treatments

Worker honeybees (*A*. *mellifera*) were provided by the Institute of Apicultural Research, Chinese Academy of Agricultural Sciences. Brood frames were placed in an incubator (34 ± 1 °C, 60 ± 5% relative humidity, darkness) after the cells were capped at approximately 9 d. Newly emerged honeybees (less than 12 h old) were assigned randomly to wooden cages (9 cm × 9 cm × 10 cm) with mesh on two sides. Each cage was fitted with a gravity feeder. Four different treatments were applied with six replications per treatment and 50 honeybees per cage. The first treatment was transgenic pollen, which was mixed thoroughly into sugar syrup (60% w/v sucrose solution) at a concentrations of 13 mg/mL (Bt-C1). Activated Cry1Ba3 toxin, provided by the Institute of Plant Protection, Chinese Academy of Agricultural Sciences, was mixed thoroughly into sugar syrup (60% w/v sucrose solution) at a concentrations of 10 µg/mL (Bt-C2). This high-concentration Cry1Ba3 toxin treatment represents the worst case scenario. Pure sugar syrup (60% w/v sucrose solution) and non-transgenic cabbage pollen were used as controls.

### Pollen consumption

For each cage, 2 g of corresponding food was supplied. This food was weighed and replaced with fresh food every 3 days for 21 days. The cages were kept in an incubator (34 ± 1 °C, 60 ± 5% relative humidity, darkness).

### Survival and weight

The honeybees were exposed to the different treatments described above for 21 days^34^. The number of surviving honeybees in each cage was recorded daily at 5:00 pm. Honeybees were considered dead when they remained completely immobile and the dead honeybees were removed from cages every day^20^. The body weight of ten randomly selected honeybees for each treatment was recorded on days 7, 14 and 21^34^.

### Measurement of detoxification enzyme activity

In each replicate, ten 7-day-old honeybees in total taken from each treatment were used for the measurement of detoxification enzyme activity. The honeybees were placed in a pre-cooled glass homogenizer and then 0.1 mol/L phosphate buffer (containing 0.1% Triton X-100, pH 8.0) was added (1:10, w/v). The mixtures were homogenized in an ice bath and then centrifuged at 10,000 × *g* for 30 min at 4 °C. The supernatant was analyzed to estimate detoxification enzyme activity. Three replicates were undertaken per treatment. The activities of glutathione-S-transferase, acetylcholinesterase and α-naphthyl acetate esterase were measured using the procedures previously described by Booth *et al*.^35^, Ellman^36^ and van Asperern^37^, respectively.

### Measurement of midgut enzyme activity

In each replicate, ten 7-day-old honeybees in total were randomly chosen from each treatment. The honeybees were dissected in an ice bath and flushed using pre-cooled NaCl (0.15 mol/L). The midguts were isolated immediately, placed in a glass homogenizer, and homogenized in an ice bath after adding 1 mL of 0.15 mol/L NaCl. The extract was then centrifuged at 15,000 × *g* for 15 min at 4 °C. The supernatant was analyzed to estimate the midgut enzyme activity. Three replicates were undertaken per treatment.

Total proteolytic enzyme activity in the midgut was measured as previously described^38^. Azocasein was used as the substrate for the proteolysis reaction and the absorption was measured at 440 nm using an 8452 A type ultraviolet spectrophotometer. The measurement of tryptase activity included assessing active alkaline trypsin-like and weak alkaline trypsin-like enzymes. Specific substrates were used to distinguish between distinct protease classes: Nα-Benzoyl-DL-arginine 4-nitroanilide hydrochloride was used to measure the active alkaline trypsin-like activity, Nα-p-tosyl-L-Arg methyl ester was used to measure the weak alkaline trypsin-like activity, and Trichlorpyr butoxyethyl ester was used to measure the chymotrypsin-like enzyme activity. The absorption was measured at 406 nm, 248 nm, and 256 nm, respectively.

### Statistical analyses

Survival was tested using the Kaplan-Meier estimator. Significant differences among all treatments for pollen consumption, weight and enzyme activity were evaluated using one-way analysis of variance (ANOVA). If significant differences were found (*P* < 0.05), multiple comparison procedures were performed with Duncans multiple-range test.

### Data availability

The datasets analysed during the current study are available in the [Supplementary Dataset] repository.

## Electronic supplementary material

Supplementary Dataset 1
