# Supplementary material for: Effects of Bt cabbage pollen on the honeybee Apis mellifera L
Source: Sci Rep. 2018 Jan 11;8:482. doi: 10.1038/s41598-017-18883-w (PMC5764958; doi:10.1038/s41598-017-18883-w)
Supplement: Supplementary file 1 — Supplementary Dataset 1 [file 41598_2017_18883_MOESM1_ESM.doc]

Effects of Bt cabbage pollen on the honeybee *Apis mellifera* L.

Dengxia Yi^1^,*, Zhiyuan Fang^2^ & Li-Mei Yang^2^,*

^1^Institute of Animal Science, Chinese Academy of Agricultural Sciences, Beijing 100193, China.

^2^Key Laboratory of Biology and Improvement of Horticultural Crops, Ministry of Agriculture, Institute of Vegetables and Flowers, Chinese Academy of Agricultural Sciences, Beijing 100081, China.

^*^Corresponding authors: D.Y. ([yidengxia@163.com](mailto:yidengxia@163.com)) and L.Y. ([yanglimeicaas@163.com](mailto:yanglimeicaas@163.com))

**The datasets analysed during the current study are provided as follows.**

**Table 1**. Number of Surviving *A*. *Mellifera* fed with Bt-C1, Bt-C2, non-Bt pollen and pure sugar syrup for 21 days.

| Food type | Day  1 | Day  2 | Day  3 | Day  4 | Day  5 | Day  6 | Day  7 | Day  8 | Day  9 | Day  10 | Day  11 | Day  12 | Day  13 | Day  14 | Day  15 | Day  16 | Day  17 | Day  18 | Day  19 | Day  20 | Day  21 |
| --- | --- | --- | --- | --- | --- | --- | --- | --- | --- | --- | --- | --- | --- | --- | --- | --- | --- | --- | --- | --- | --- |
| Bt-C1 | 300 | 297 | 297 | 290 | 280 | 275 | 261 | 253 | 252 | 248 | 237 | 229 | 226 | 225 | 221 | 216 | 214 | 211 | 211 | 209 | 206 |
| Bt-C2 | 300 | 300 | 297 | 289 | 277 | 271 | 264 | 259 | 246 | 242 | 241 | 241 | 229 | 226 | 226 | 223 | 211 | 203 | 199 | 197 | 194 |
| non-Bt | 300 | 300 | 294 | 289 | 275 | 266 | 263 | 261 | 251 | 245 | 240 | 237 | 230 | 229 | 225 | 219 | 218 | 215 | 211 | 202 | 199 |
| Syrup | 299 | 299 | 295 | 292 | 278 | 268 | 266 | 254 | 244 | 244 | 242 | 233 | 231 | 221 | 218 | 214 | 210 | 209 | 206 | 199 | 198 |

**Table 2**. Three-day cumulative quantify of food consumed by *A*. *Mellifera* subjected to chronic exposure to Bt-C1, Bt-C2, non-Bt pollen and pure sugar syrup during a 21-day oral exposure (unit: mg).

| Food type | Days | Repeat 1 | Repeat 2 | Repeat 3 | Repeat 4 | Repeat 5 | Repeat 6 |
| --- | --- | --- | --- | --- | --- | --- | --- |
| Bt-C1 | 1-3 | 9.26 | 10.3 | 7.65 | 10.25 | 13.47 | 7.98 |
|  | 4-6 | 10.73 | 10.65 | 6.01 | 10.92 | 7.11 | 10.96 |
|  | 7-9 | 5.65 | 6.35 | 10.15 | 8.7 | 7.2 | 9.93 |
|  | 10-12 | 6.63 | 9.32 | 5.11 | 8.19 | 4.69 | 7.62 |
|  | 13-15 | 3.66 | 5.92 | 8.59 | 4.96 | 8.03 | 2.23 |
|  | 16-18 | 6.52 | 4.51 | 2.94 | 5.65 | 2.78 | 4.32 |
|  | 19-21 | 2.93 | 1.55 | 1.1 | 1.16 | 1.23 | 3.13 |
|  | Sum | 45.38 | 48.6 | 41.55 | 49.83 | 44.51 | 46.17 |
|  |  |  |  |  |  |  |  |
| Bt-C2 | 1-3 | 7.52 | 11.59 | 9.91 | 10.03 | 6.05 | 10.22 |
|  | 4-6 | 6.22 | 10.74 | 7.88 | 12.15 | 10.96 | 9.94 |
|  | 7-9 | 8.96 | 6.3 | 4.78 | 9.72 | 7.2 | 10.45 |
|  | 10-12 | 7.55 | 4.99 | 8.13 | 7.49 | 10.65 | 8.66 |
|  | 13-15 | 2.7 | 2.88 | 6.03 | 5.02 | 4.15 | 6.18 |
|  | 16-18 | 3.3 | 4.23 | 1.99 | 4.45 | 1.67 | 3.86 |
|  | 19-21 | 4.26 | 2.01 | 1.33 | 0.58 | 2 | 0.89 |
|  | Sum | 40.51 | 42.74 | 40.05 | 49.44 | 42.68 | 50.2 |
|  |  |  |  |  |  |  |  |
| non-Bt | 1-3 | 12.97 | 9.68 | 8.36 | 10.12 | 10.09 | 7.3 |
|  | 4-6 | 6.89 | 7.02 | 12.16 | 10.97 | 8.95 | 11.21 |
|  | 7-9 | 6.06 | 10.03 | 8.63 | 4.3 | 9.05 | 7.25 |
|  | 10-12 | 7.59 | 4.58 | 6.59 | 8.32 | 9.11 | 5.92 |
|  | 13-15 | 6.87 | 6.02 | 5.09 | 2.43 | 6.15 | 2.94 |
|  | 16-18 | 4.26 | 2.81 | 3.92 | 6.85 | 1.61 | 1.57 |
|  | 19-21 | 1.36 | 3.48 | 1.13 | 2.89 | 0.55 | 0.94 |
|  | Sum | 46 | 43.62 | 45.88 | 45.88 | 45.51 | 37.13 |
|  |  |  |  |  |  |  |  |
| Syrup | 1-3 | 6.25 | 10.28 | 9.89 | 9.94 | 11.36 | 7.34 |
|  | 4-6 | 9.15 | 6.88 | 8.86 | 10.2 | 11.16 | 7.02 |
|  | 7-9 | 5.66 | 6.78 | 10.12 | 6.09 | 9.85 | 5.97 |
|  | 10-12 | 8.19 | 4.22 | 5.03 | 9.88 | 6.23 | 5.99 |
|  | 13-15 | 2.21 | 5.77 | 6.97 | 4.33 | 6.42 | 5.86 |
|  | 16-18 | 4.88 | 3.69 | 4.04 | 2.17 | 2.05 | 3.64 |
|  | 19-21 | 0.36 | 1.22 | 3.03 | 2.01 | 0.96 | 1.27 |
|  | Sum | 36.7 | 38.84 | 47.94 | 44.62 | 48.03 | 37.09 |

**Table 3**. Body weight of *A*. *Mellifera* fed with Bt-C1, Bt-C2, non-Bt pollen and pure sugar syrup for 21 days (unit: mg).

| Days | Food type | Repeat 1 | Repeat 2 | Repeat 3 | Repeat 4 | Repeat 5 | Repeat 6 |
| --- | --- | --- | --- | --- | --- | --- | --- |
| Day 7 | Bt-C1 | 102.53 | 96.77 | 98.29 | 90.55 | 85.71 | 93.67 |
|  | Bt-C2 | 79.42 | 105.38 | 88.75 | 95.4 | 100.24 | 83.2 |
|  | non-Bt | 103.25 | 104.63 | 88.76 | 99.53 | 85.98 | 100.55 |
|  | Syrup | 93.25 | 100.56 | 77.25 | 78.9 | 94.5 | 86.73 |
|  |  |  |  |  |  |  |  |
| Day 14 | Bt-C1 | 131.78 | 136.2 | 142.75 | 112.88 | 126.86 | 119.78 |
|  | Bt-C2 | 144.66 | 132.73 | 138.63 | 128.68 | 143.49 | 140.21 |
|  | non-Bt | 133.45 | 140.55 | 140.48 | 137.25 | 125.33 | 134.58 |
|  | Syrup | 128.77 | 136.69 | 131.69 | 129.77 | 132.56 | 135.63 |
|  |  |  |  |  |  |  |  |
| Day 21 | Bt-C1 | 126.93 | 123.17 | 109.17 | 110.98 | 115.42 | 119.88 |
|  | Bt-C2 | 100.77 | 113.55 | 104.98 | 97.63 | 110.85 | 116.79 |
|  | non-Bt | 105.46 | 109.53 | 118.62 | 115.67 | 110.49 | 117.82 |
|  | Syrup | 99.95 | 103.22 | 118.75 | 121.34 | 119.45 | 108.98 |

**Table 4. The activities of three detoxification enzymes in *A*. *Mellifera* fed with Bt-C1, Bt-C2, non-Bt pollen and pure sugar syrup for 7 days (unit: mmol·L^-1^·mg^-1^·min^-1^).**

| detoxification enzymes | Food type | Repeat 1 | Repeat 2 | Repeat 3 |
| --- | --- | --- | --- | --- |
| α-naphthylacetate esterase | Bt-C1 | 0.036 | 0.029 | 0.038 |
|  | Bt-C2 | 0.048 | 0.033 | 0.04 |
|  | non-Bt | 0.037 | 0.029 | 0.044 |
|  | sugar syrup | 0.036 | 0.033 | 0.036 |
|  |  |  |  |  |
| Acetylcholinesterase | Bt-C1 | 0.058 | 0.053 | 0.054 |
|  | Bt-C2 | 0.057 | 0.048 | 0.052 |
|  | non-Bt | 0.062 | 0.044 | 0.049 |
|  | sugar syrup | 0.047 | 0.063 | 0.049 |
|  |  |  |  |  |
| Glutathione-S-transferase | Bt-C1 | 0.016 | 0.014 | 0.014 |
|  | Bt-C2 | 0.012 | 0.017 | 0.018 |
|  | non-Bt | 0.01 | 0.015 | 0.017 |
|  | sugar syrup | 0.011 | 0.014 | 0.013 |

**Table 5. The activities of total proteolytic enzyme, active alkaline trypsin-like enzyme, weak alkaline trypsin-like enzyme and chymotrypsin-like enzyme in *A*. *Mellifera* fed with Bt-C1, Bt-C2, non-Bt pollen and pure sugar syrup for 7 days (μmol·mg^-1^·min^-1^).**

| Midgut enzyme | Food type | Repeat 1 | Repeat 2 | Repeat 3 |
| --- | --- | --- | --- | --- |
| Total proteolytic enzyme activity | Bt-C1 | 0.018 | 0.012 | 0.021 |
|  | Bt-C2 | 0.009 | 0.017 | 0.018 |
|  | non-Bt | 0.016 | 0.018 | 0.015 |
|  | sugar syrup | 0.02 | 0.011 | 0.016 |
|  |  |  |  |  |
| Active alkaline trysin-like enzyme activity | Bt-C1 | 0.022 | 0.024 | 0.019 |
|  | Bt-C2 | 0.026 | 0.024 | 0.023 |
|  | non-Bt | 0.027 | 0.024 | 0.024 |
|  | sugar syrup | 0.025 | 0.017 | 0.016 |
|  |  |  |  |  |
| Weak alkaline trysin-like enzyme activity | Bt-C1 | 0.67 | 0.78 | 0.6 |
|  | Bt-C2 | 0.78 | 0.82 | 0.83 |
|  | non-Bt | 0.79 | 0.84 | 0.72 |
|  | sugar syrup | 0.69 | 0.66 | 0.81 |
|  |  |  |  |  |
| Chymotrypsin-like enzyme activity | Bt-C1 | 0.14 | 0.18 | 0.16 |
|  | Bt-C2 | 0.12 | 0.19 | 0.18 |
|  | non-Bt | 0.21 | 0.19 | 0.22 |
|  | sugar syrup | 0.24 | 0.19 | 0.22 |
